# Childhood Trauma, the Combination of MAO-A and COMT Genetic Polymorphisms and the Joy of Being Aggressive in Forensic Psychiatric Patients

**DOI:** 10.3390/brainsci11081008

**Published:** 2021-07-30

**Authors:** Michael Fritz, Franziska Rösel, Hannah Dobler, Judith Streb, Manuela Dudeck

**Affiliations:** Department of Forensic Psychiatry and Psychotherapy, Ulm University, 89312 Guenzburg, Germany; franziska.roesel@uni-ulm.de (F.R.); hannah.dobler@t-online.de (H.D.); Judith.Streb@bkh-guenzburg.de (J.S.); Manuela.Dudeck@bkh-guenzburg.de (M.D.)

**Keywords:** forensic patients, appetitive aggression, reactive aggression, genetic polymorphism, MAO-A, COMT

## Abstract

Aggression and violent offenses are common amongst forensic psychiatric patients. Notably, research distinguishes two motivationally distinct dimension of aggression–instrumental and reactive aggression. Instrumental aggression comprises of appetitive, goal-directed aggressive acts, whereas reactive aggression consists of affective, defensive violence with both their biological basis remaining largely unknown. Childhood trauma and functional genetic polymorphisms in catecholamines converting enzymes, such as mono-amino-oxidase A (MAO-A) and catechol-o-methyltransferase (COMT) have been suggested to augment an aggressive behavioral response in adulthood. However, it warrants clarification if these factors influence one or both types of aggression. Furthermore, it remains elusive, if having a combination of unfavorable enzyme genotypes and childhood maltreatment further increases violent behavior. Hence, we set out to address these questions in the current study. First, analysis revealed an overall marginally increased frequency of the unfavorable MAO-A genotype in the test population. Second, each gene polymorphisms together with a traumatic childhood significantly increased the AFAS (Appetitive and Facilitative Aggression Scale) scores for both reactive and appetitive aggression. Third, having a combination of both disadvantageous genotypes and a negative childhood served as a minor positive predictor for increased reactive aggression, but had a strong influence on the joy of being aggressive.

## 1. Introduction

Aggression is a complex behavior that essentially aims to injure people or damage objects and is intricately linked to human evolution. Commonly, psychologists differentiate two subtypes of aggression-reactive and instrumental aggression [1]. Reactive aggression (e.g., an impulsive, affective defense) is primarily believed to be an adaptive response if displayed in an appropriate context (e.g., fighting for one’s life); however, if exaggerated or occurring often in inappropriate contexts, it quickly becomes maladaptive behavior [2]. Another type of maladaptive violence is instrumental (or premeditated) aggression, which serves as an intentional tool to reach specific goals (e.g., to obtain a reward such as social status or material goods) and is not driven by anger. Recent research coined an additional subcategory of maladaptive aggression called “appetitive aggression” [3,4]. Appetitive aggression describes a hedonically driven motivation for violence, which underlies for example hunting and extreme acts of violence, such as sadistically oriented, anticipated murder or massacres. This joy of being aggressive was previously assumed to be only seen in psychopaths but can occur under certain circumstances (e.g., situational polarization, dehumanization of political enemies, ethicizes, etc.) in previous peaceful, law-obeying individuals [5]. These forms of maladaptive aggression may be the consequence of excessive activation of neuronal reward circuits and especially the neurotransmitter dopamine [6]. In other words, if human beings learn that aggression is a successful tool to reach goals, they will continue to use it. However, there exist additional factors that may contribute to the transition from adaptive to maladaptive aggression; some that are of environmental and genetic nature.

The single most reported environmental factor related to augmented aggressive and antisocial behavior is a history of maltreatment (e.g., emotional, sexual, or physical abuse) during childhood [7,8,9]. A history of abuse also predicts emotional dysregulation, affective lability, and socially inappropriate emotional expressions [10]. Furthermore, physical abuse during childhood is associated with increased reactive aggression among violent offenders [11]. Finally, appetitive aggressive, antisocial behavior is highly correlated with adverse childhood experiences and exaggerated in forensic psychiatric patients [12,13].

Genetic factors contributing to maladaptive aggression center around dopaminergic or (to the wider extend) catecholaminergic transmission in the central nervous system. Here, primarily two enzymes regulate dopamine metabolism in the synaptic cleft-monoamine oxidase-A (MAO-A) and catechol-O-methyltransferase (COMT).

The gene coding for MAO-A is located at the short arm of the X chromosome with a variable tandem repeat polymorphism in the promoter region of MAO-A. This functionally different polymorphism consists of a 30-base pair repeated sequence existing in 2, 3, 3.5, 4 or 5 repeats (R). 3.5 R, 4 R or 5 R variants are called MAO-A high, whereas the 2 R and 3 R versions are known as MAO-A low. Carriers of the MAO-A high variant have 2 to 10 times increased enzymatic activity and hence a much larger monoamine metabolism [14]. A number of studies [15,16] were able to ink the MAO-A low variant to exaggerated aggressive behavior.

COMT activity is controlled by the COMT gene located on the chromosome band 22q11.2 [17]. Here, a particular single nucleotide polymorphism, the Val108/158Met polymorphism, where the amino acid valine is replaced by methionine has been the center of scientific attention [18]. This substitution can lead to three different gene expressions (Met/Met, Val/Met and Val/Val). Human beings with the Val/Val variant have a 2 to 4 times higher COMT enzyme activity than subjects with the expression Met/Met [19]. Regarding aggressive behavior, Val/Val carriers scored in a study on suicide attempters higher on trait anger (i.e., disposition to experience anger) than test persons with the two other genotypes [20]. Additionally, the Val allele has been found to be linked to explicitly aggressive symptoms of conduct disorder in youth [21]. Finally, Caspi and Colleagues [22] demonstrated that young Val/Val homozygotes with ADHD expressed more behavior linked to conduct disorder, were more aggressive, and were more likely to be convicted of criminal offenses.

Intriguingly, to date there are no studies published investigating the interaction between adverse childhood experiences, genotypic variants of COMT and MAO-A, and aggression in forensic psychiatric inpatients. Furthermore, to the best of our knowledge, there exists only one study on unfavorable genotypes of both metabolic enzymes measured in a single individual and related to traumatic early life events [23]. Notably, this study focused on a different COMT mutation, where alanine is substituted with serine.

Hence, the aim of the present study was to first assess the frequency of COMT and MAO-A variants as well as traumatic childhood experiences in forensic psychiatric patients. Secondly, to evaluate the influence of a COMT x MAO-A variants × traumatic childhood experiences interaction on both reactive and appetitive aggression.

## 2. Materials and Method

### 2.1. Ethical Approval Statement

The study was conducted in accordance with the Declaration of Helsinki and approved by the ethical review board of the University of Ulm (No. 497/18).

### 2.2. Participants

A total of 64 inpatients were recruited from the forensic psychiatry departments of the district hospitals Günzburg (*n* = 44) and Kaufbeuren (*n* = 20), Bavaria, Germany. Three patients had to be excluded from the study, since no DNA for genotyping could be isolated from their salvia samples. All remaining 61 patients were male, 18 years or older, had sufficient knowledge of the German language, and were convicted for crimes in the context of drug addiction according to section 64 of the German Criminal Code. The only study exclusion criterion was a clinically manifested thought disorder (disturbances in cognition), although no such case was reported.

### 2.3. Data and Saliva Sample Acquisition

Data and saliva sample collection was done by scientifically trained personnel visiting the different infirmaries. Here, information materials about study aims and procedures were handed out and any questions or concerns raised were addressed directly. If patients decided to participate, either an appointment was made or data acquisition and saliva sampling were conducted immediately. Local examination and conference rooms were used for the evaluation. Data collection lasted between 30 and 50 min. First, each participant had to sign three different declarations of consent (study participation, German privacy protection act, and sampling of body fluids). Thereafter, subjects conducted a total of four cheek swabs under the supervision of the scientific personnel and were then asked to procced answering the questionnaires. The survey could be terminated without reason and participation was not remunerated.

### 2.4. Questionnaires

#### 2.4.1. Sociodemographic, Clinical, and Forensic Characteristics

Participants were asked to provide information regarding their age, level of education, current diagnosis, index criminal offenses, and length of the current treatment in months.

#### 2.4.2. Assessment of Adverse Childhood Experiences

Adverse childhood experiences were assessed with the German version (“Belastende Kindheitserfahrungen”, KERF, [24]) of the Maltreatment and Abuse Chronology of Exposure Scale [25]. KERF is a retrospective self-rating questionnaire containing 75 items, which are assigned to the following 10 subscales: parental physical abuse (six items), parental verbal abuse (four items), parental nonverbal emotional abuse (five items), sexual abuse (12 items), emotional neglect (10 items), physical neglect (six items), witnessed physical violence toward parents (eight items), witnessed violence toward siblings (seven items), peer emotional violence (four items) and peer physical violence (four items). Each item (example question: “Did your parents lock you in a closet, storage area, basement, garage or other, perhaps even very narrow, dark location?” [authors’ translation]) can be answered “yes” or “no”; “yes” is assigned a value of 1 and “no” a value of 0 and the values are summed for each subscale. The authors of the KERF scale provide cut-off values for each subscale (see [24]). In our study, participants were categorized as having been abused if their sum values were above the cut-off value in at least one of the subscales. The KERF scale was validated with the Childhood Trauma Questionnaire [26] and correlated strongly with both the total score and the subscale scores. The authors do not provide information on reliability.

#### 2.4.3. Assessment of Appetitive and Reactive Aggression

We assessed appetitive and reactive aggression with the German version of the Appetitive and Facilitative Aggression Scale (AFAS; [4]). This 30-item scale has 15 items each on appetitive aggression (example question: “Did you provoke others simply because it was fun for you?” [authors’ translation]) and reactive aggression (example question: “Did you step on an object or throw it around because you were frustrated?” [authors’ translation]). The items are answered on a 5-point Likert scale ranging from 0 (never) to 4 (very often). We summed the items to calculate a score for appetitive aggression and one for reactive aggression, each ranging from 0 to 60, whereby a higher score indicated a higher level of aggression. According to Augsburger et al. [3] the reliability of the scale is good, with a Cronbach’s α of 0.87 for reactive aggression and 0.78 for appetitive aggression.

### 2.5. Salvia Sampling and Genotyping

Saliva samples were collected with cotton swabs approved for forensic use (102 × 15 mm; Sarstedt AG; Nümbrecht). Once the sample was collected, each swab was stored in a sampling tube at room temperature until collectively sent to Berlin, Germany. DNA isolation and genotyping were performed commercially at the IMD Institute for medical diagnostics Berlin-Potsdam GbR (IMD Berlin MVZ). For DNA isolation a QIAamp DNA Mini-Kit (Qiagen) was used. Subsequently, COMT-genotyping was done with a Light SNiP rs4680-Kit from TibMolBiol, Berlin. For MAOA-Genotyping a polymerase chain reaction (PCR) procedure (3130 Genetic Analyzer (Fa. Applied Biosystems)) was used (Forward-Primer tagtaaaacgacggccagtcagcctgaccgtggagaagg; Reverse-Primer tacaggaaacagctatgacaacggacgctccattcgga).

### 2.6. Statistics

SPSS Statistics for Windows (IBM Corp. Released 2020. IBM SPSS Statistics for Windows, Version 26.0. Armonk, NY, USA: IBM Corp.) was used for analysis. First, descriptive statistics (mean, standard deviation, absolute and relative frequency) were generated. The observed frequencies of MAO-A high/low as well as COMT-Met/Met, -Val/Met and -Val/Val were compared to their estimate rate of occurrence via an X^2^-Test. A generalized linear model (GZLM) was generated to analyze any effects of childhood events and genotypes. When predicting the dependent variables reactive and appetitive aggression the model type was specified as linear (i.e., normal distribution and identity as link function). As predictors served genotypes [Val/Val AND MAO-A low (2), Val/Val or MAO-A low (1), Val/Met or Met/Met AND MAO-A high (0)] and adverse childhood experiences [yes (1), no (0)], as well as their interaction. To estimate the covariance matrix a robust estimator (Huber-White sandwich estimator) was chosen. *p*-values of equal or less than 5% (*p* ≤ 0.05) were considered statistically significant.

## 3. Results

Table 1 shows a summary of the descriptive statistics of the patient population. Age and length of the current treatment are displayed as mean ± standard deviation. Level of education, main and secondary diagnosis, and index crimes are shown as actual numbers and percentage of the total test population.

### 3.1. Frequency Distribution of MAO-A Variants across the Sample

A total of 27 participants (44%) tested positive for the “MAO-A low” gene composition, whereas 34 patients (56%) had the “high” activity variant. It is worthwhile noting, that these numbers deviate from an expected distribution within a normal population towards a more frequent occurrence [27]. However, the effect is currently not statistically significant (Table 2; *X*^2^(1) = 2.300, *p* = 0.06).

### 3.2. Frequency Distribution of COMT Variants across the Sample

In the current patient sample the number of detected variants for COMT were *n* = 19 Val/Val (31%), *n* = 29 Val/Met (48%), and *n* = 13 Met/Met (21%). These numbers are in accordance with the expected distribution in the literature (Table 3; *X*^2^(1) = 1.328, *p* = 0.52; [28]).

### 3.3. Distribution of Genotype Combination and Childhood Experiences amongst the Test Group

To address the question, if having two risky genetic predispositions increases reactive or appetitive aggression, we first calculated the rate of occurrence of combined favorable and unfavorable MAO-A/COMT sequences in combination with childhood experiences. In total, around 70% (*n* = 43) of all patients reported adverse childhood experiences with no distribution difference amongst the genetic subgroups observed (*X*^2^(2) = 0.159, *p* = 0.923, Table 4).

### 3.4. The Effect of Childhood Experiences and MAO-A/COMT Gene Variant Combination on Reactive and Appetitive Aggression

**Figure 1:**
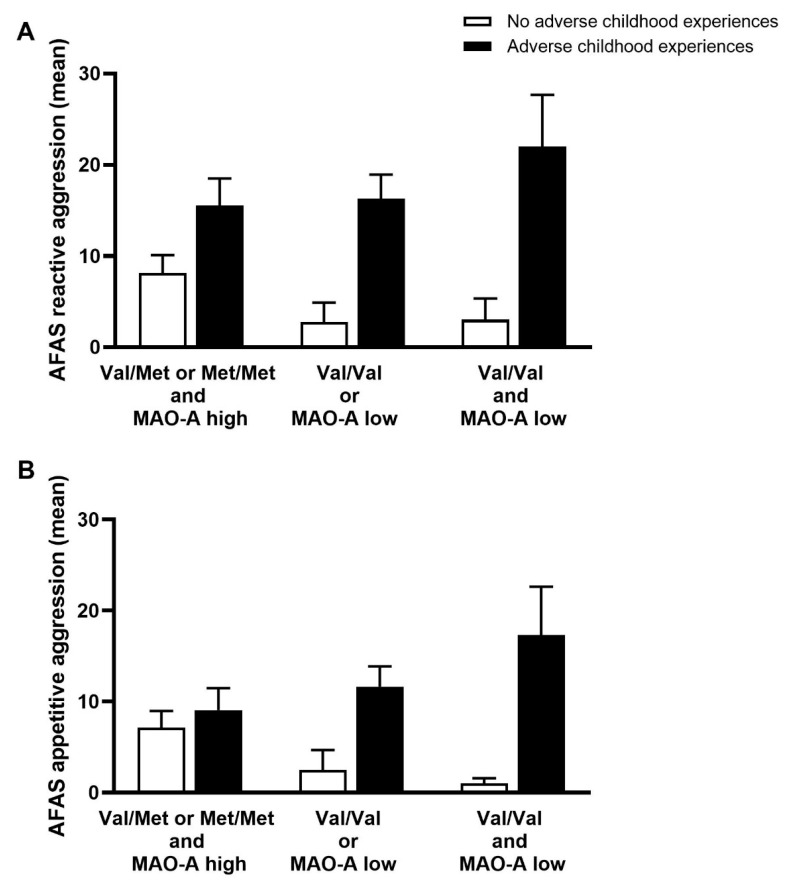
Generalized linear model. (**A**) generalized linear model revealed a significant main effect of both genotype and childhood experiences on reactive aggression; (**B**) generalized linear model for the influence of genotype and childhood experiences on appetitive aggression.

The generalized linear model revealed a significant main effect of both genotype and childhood experiences (Figure 1A). First, MAO-A/COMT genotype variants had a significant effect on mean AFAS scores in the absence of childhood abuse (*b* = −3.226, *SE*(*b*) = 1.37, *p* = 0.019). In such a case, mean AFAS scores for reactive aggression were 3.23 (Val/Val or MAO-A low) and 6.45 (Val/Val and MAO-A low) points lower than in the Val/Met or Met/Met and MAO-A group. Second, adverse childhood experiences led to a strong increase in mean AFAS scores for reactive aggression (*b* = 7.169, *SE*(*b*) = 3.16, *p* = 0.023). Here, the Val/Val and MAO-A low group had an averagely increased mean of 7.17 points. Third, the interaction MAO-A/COMT * adverse childhood experiences turned out to a be marginally significant predictor (*b* = 5.932, *SE*(*b*) = 3.07, *p* = 0.05).

In Figure 1B another generalized linear model for the influence of genotype and childhood experiences on appetitive aggression was calculated. Like in the case of reactive aggression, the main effect of COMT/MAO-A gene variants turned out to be significant (*b* = −3.286, *SE*(*b*) = 1.06, *p* = 0.002). In the absence of adverse childhood experiences, Val/Val or MAO-A low and Val/Val and MAO-A low groups scored 3.27 and 6.57 points lower on the AFAS appetitive aggression scale than patients with Val/Met or Met/Met and MAO-A high. However, adverse childhood experiences themselves had no influence on appetitive aggression (*b* = 1.719, *SE*(*b*) = 2.72, *p* = 0.528). Yet, the interaction between COMT/MAO-A * adverse childhood experiences formed a highly significant positive predictor (*b* = 7.122, *SE*(*b*) = 2.66, *p* = 0.007). In other words, if patients from the Val/Val or MAO-A low and Val/Val and MAO-A low groups experienced maltreatment as children, their AFAS appetitive aggression means were 7.12 and 14.24 higher, respectively.

## 4. Discussion

In summary, our study demonstrates that there is a strong tendency of an overrepresentation of the MAO-A low genotype within the patient population. This is not the case for for the COMT polymorphism. However, and most intriguingly, an interaction between the COMT Val/Val × MAO-A low × adverse childhood experiences serves as a strong predictor for the joy of being aggressive and as a weak predictor for an augmented reactive aggressive behavioral response.

Undoubtedly, a great body of research underlines the impact of adverse childhood experiences on behavioral patterns during adulthood. Such individuals tend to have more physical and mental health problems and are generally at risk for a premature death [29,30]. Furthermore, childhood trauma, aggressive behavior, and the increased probability of committing a crime are strongly associated with one another [11,31,32,33,34], with a significant amount of forensic psychiatric inpatients describing traumatic childhood experiences [35].

When it comes to the role of COMT in the context of gene mutation, adverse childhood events, and aggression scientific results are not as clear. On one hand, the Met-allele has been linked to aggressive, hostile, and violent behavior in schizophrenia patients [36,37,38]. Young adults carrying the Met/Met variant had higher levels of both physical aggression and relational aggression [39]. However, the Val/Val variant was explicitly associated with aggressive symptoms of conduct disorder in youth [21]. In line with this, Val/Val homozygotes were more aggressive and were more likely to be convicted of criminal offenses in youth with ADHD [22].

For MAO-A on the other hand, the picture is much more consistent. The groundbreaking study of Caspi and colleagues [7] demonstrated that a genotype alone was not associated with antisocial behavior but combined with childhood maltreatment the MAO-A low variant made subjects more susceptible to react aggressive. Additional studies were able to confirm this observation [40,41,42]. Intriguingly, even across species in non-human primates, such an adverse effect of MAO-A low in combination with stressful early-life experiences could be seen [43]. Further research revealed even a connection between the MAO-A low variant and morphological and functional changes in the human brain (for a detailed review see [44]).

From a metabolic point of view, the observation made in our study could explain the inconsistencies regarding the two catecholamine enzymes. COMT is the upstream enzyme that metabolizes Dopamine in the synaptic cleft to 3-Methoxytyramine (3-MT; [45]). 3-MT is then further processed into homovanillic acid (HVA) by MAO-A, a product which is normally excreted in the urine [46]. Until quite recently, 3-MT had not received much attention as it was thought to have no biological function. Yet, in 2010, work from Sotnikova and colleagues [45] demonstrated that 3-MT is an agonistic neuromodulator of the Trace amine-associated receptor 1 (TAAR1). TAAR1 is an intracellular amine-activated Gq protein-coupled receptor (GPCR) that is expressed throughout the body but is also present in the presynaptic axon terminal of monoamine neurons [47]. It plays a significant role in regulating neurotransmission in dopaminergic, norepinephrinergic, and serotonergic neurons in the CNS [48]. Since COMT Val/Val carrier have a 2 to 4 time higher enzymatic activity [19], consequently a larger amount of 3-MT has to accumulate in the synaptic cleft upon dopamine release. If this accumulation is met by MAO-A low activity variant, MAO-A cannot compensate to balance the augmented accumulation. Following, 3-MT can bind in greater amount to the TAAR1 receptor potentially causing changes in activity patterns on monoaminergic neurons. In the light of such thoughts, it seems intriguingly that perpetrators influencing aggressive behavior might not solidly be the monoamines. However, further research must be conducted to proof this hypothesis. For the time being, a double COMT and MAO-A gene analysis seems the best way forward for future studies. Yet, it should be clearly stated, that even such a combination of unfavorable genotypes and changes in neurotransmitter levels cannot solidly be made responsible for aggression. Human behavior is always the consequence of a gene and environment interaction [49]. Variations of genes may influence the probability of a certain behavior to occur, but do not exclusively determine it [50]. Dylan B. Jackson [51] summarized this observation perfectly: “The effect of genetic polymorphism on antisocial behavior is at least a partial product of multiple genes, with certain alleles either increasing or decreasing the probability of a particular phenotype occurring or affecting the distribution of the trait.”

Finally, there are four limiting factors in this study. First, a cross-sectional study design neither provides evidence about the direction of the interaction nor if such an interaction is of causal nature. Second, some of the effects may have been stronger, if a larger patient population would have been screened. Unfortunately, forensic psychiatric patients are a small and legally protected group. Thus, we were not able to provide a larger number of participants. Third, the study did not provide an assessment of psychopathy as an influencing factor, since the Psychopathy Check list (PCL) is not used routinely in the treatment facilities. Hence, it cannot be ruled out that the COMT Val/Val and MAO-A low genotype combination and the accompanying effect on the AFAS is biased by psychopathic traits. Ultimately, the study did not control for the ethnic background of the test group. There may be inter-ethnic differences regarding the effects of genetic variants. For example, Stetler and colleagues [52] demonstrated a connection between MAO-A low and violent crimes only in Caucasians, but not African Americans.

## 5. Conclusions

The central findings of this study are that the MAO-A low variant is more common than expected in a forensic psychiatric patient population. Furthermore, a combination of MAO-A low and COMT Val/Val variants together with traumatic childhood experiences increase both reactive and appetitive aggression.

## Figures and Tables

**Table 1:** Descriptive statistics of the patient population (SD = standard deviation; *n* = number).

	Mean (SD)
Age (years)	34.00 (9.70)
Length of treatment (Months)	11.35 (12.24)
	***n* (percentage)**
**Level of education**	
No formal Diploma	12 (19%)
Middle School Diploma	29 (45%)
Secondary School Diploma	17 (27%)
High School Diploma	6 (9%)
**Main Diagnosis (ICD-10)**	
F1 (all drug-related disorders)	62 (97%)
F6 (personality disorders)	2 (3%)
**Secondary Diagnosis (ICD-10)**	
F3 (affective disorders)	5 (8%)
F6 (personality disorders)	14 (22%)
Others	3 (5%)
**Index crime**	
Murder/Manslaughter	2 (3%)
Robbery	7 (11%)
Aggravated Battery	11 (17%)
Sexual Assault	1 (2%)
Fraud/Theft	11 (17%)
Violation of the Narcotics Act	29 (45%)
Others	3 (5%)

**Table 2:** Comparison of the MAO-A gene variants distribution in terms of expected versus observed distribution (Low: MAO-A-Gen-Allele 3 R, High: MAO-A-Gen-Allele 4 R, Residuum: Difference between the anticipated number versus the observed number of gene expression variants relative to the sample size).

	Actual Number	Expected Distribution	Expected Number	Residuum
Low	27	35%	21.3	5.7
High	34	65%	39.7	−5.6

**Table 3:** Comparison of the COMT-Val/Val, Val/Met, Met/Met gene variants distribution in terms of expected versus observed distribution (Val = Valin, Met = Methionine, Residuum: Difference between the anticipated number versus the observed number of gene expression variants relative to the sample size).

	Actual Number	Expected Distribution	Expected Number	Residuum
Val/Val	19	25%	15.3	3.8
Val/Met	29	50%	30.5	−1.5
Met/Met	13	25%	15.3	−2.2

**Table 4:** Occurrence of the “double risk” genotype Val/Val and MAO-A low, the “single risk” genotype Val/Val or MAO-A low and the “non-risk’ genotype Val/Met or Met/Met and MAOA-A high in the test group.

	Adverse Childhood Experiences	No Adverse Childhood Experiences	Total
Val/Val and MAO-A low	7	3	10
Val/Val or MAO-A low	19	7	26
Val/Met or Met/Met and MAO-A high	17	8	25
Total	43	18	61
